# Including the voice of people living with viral hepatitis: lessons learned from Japan to accelerate progress towards global hepatitis elimination

**DOI:** 10.1186/s41182-021-00371-7

**Published:** 2021-10-01

**Authors:** Atsuko Yonezawa, Rebecca Grant, Yusuke Shimakawa

**Affiliations:** 1 Japan Hepatitis Council, 4-27-5-201 Shimo-ochiai, Shinjuku-ku, Tokyo, 161-0033 Japan; 2 Unité d’Épidémiologie des Maladies Émergentes, Institut Pasteur, 25-28 rue du Dr Roux, 75015 Paris, France; 3 Sorbonne Université, Paris, France

## Abstract

Despite the growing momentum created by the WHO for eliminating viral hepatitis as a public health threat by 2030, the global response is still slow and more actions are needed to meet the elimination goals, especially in low-income and middle-income countries. Japan is one of a handful of countries currently on track to achieve the WHO hepatitis elimination targets by 2030. To better understand the successful control of viral hepatitis in Japan, it is important to recognize the role of the patient association for viral hepatitis, known as the “Japan Hepatitis Council”, which celebrates its 50th anniversary in 2021. The greatest impact of the Japan Hepatitis Council has been in achieving wider access to antiviral treatments for viral hepatitis. The example of Japan and the Japan Hepatitis Council highlights the need for the engagement of civil society and patient groups to ensure equitable access to hepatitis services and to accelerate the global hepatitis elimination.

In May 2016, the World Health Assembly endorsed the Global Health Sector Strategy on viral hepatitis, with a goal to globally eliminate hepatitis B virus (HBV) and hepatitis C virus (HCV) infections as a public health threat by 2030. This is defined as a 90% decrease in incidence of chronic HBV and HCV infections and a 65% decrease in HBV- and HCV-related mortality compared to the 2015 baseline [1]. Since then, enormous efforts have been devoted to an effective public health response in many Member States. The number of countries that have launched national hepatitis strategic plans has dramatically increased from 17 in 2012 to 124 by 2019 [2, 3].

An interim WHO report on the global progress on the elimination of viral hepatitis, however, revealed a slow progress for the majority of the impact indicators in 2019 compared to 2015: number of people living with chronic HBV infection (257 million in 2015 and 296 million in 2019), number of people living with chronic HCV infection (71 million in 2015 and 58 million in 2019), number of HBV-related deaths (887,000 in 2015 and 820,000 in 2019), and number of HCV-related deaths (399,000 in 2015 and 290,000 in 2019) [3, 4]. This slow response at a global level may be related to the fact that the majority of areas with the greatest burden of viral hepatitis are low-income and middle-income countries and often have the lowest coverage for hepatitis services including the prevention, testing, and treatment [3, 5].

According to the estimates made by the Center for Disease Analysis Foundation, Japan is one of a handful of countries currently on track to achieve the elimination targets for both HBV and HCV infections by 2030 [6]. Large-scale nationwide serosurveys of first-time blood donors in Japan indicate that the prevalence of HBsAg has been steadily decreasing over time: 0.63% in 1995–2000, 0.31% in 2001–2005, and 0.20% in 2007–2011. A similar reduction has been observed in the prevalence of antibody to HCV (anti-HCV): 0.49%, 0.26%, and 0.16%, respectively [7]. To better understand the successful control of viral hepatitis in Japan, it is essential to recognize the important role of local civil societies, particularly that of the patient association for viral hepatitis (currently known as the Japan Hepatitis Council, JHC), which this year celebrates its 50th anniversary.

In 1971, the JHC, initially named as the “Hepatitis Association”, was established by Dr Hiromichi Nakajima in Tokyo (Fig. 1). He was a medical doctor, who himself suffered from chronic non-A non-B hepatitis. After seeing many chronic hepatitis patients who lived in deep fear of its unclear aetiology, lack of effective therapy, and a social stigma attached to “the disease of alcoholics”, Dr Nakajima started an association with six other hepatitis patients to offer peer counselling. They then submitted a formal request to the Ministry of Health and Welfare and to the Japanese Society of Hepatology to lead the development of diagnostics and therapies for chronic hepatitis, elucidate the disease burden with chronic hepatitis and cirrhosis in Japan, and take measures to end stigma and discrimination. In the 1980s, these grassroots activities were expanded across Japan, leading to the establishment of more than 30 local patient associations for viral hepatitis.

**Fig. 1:**
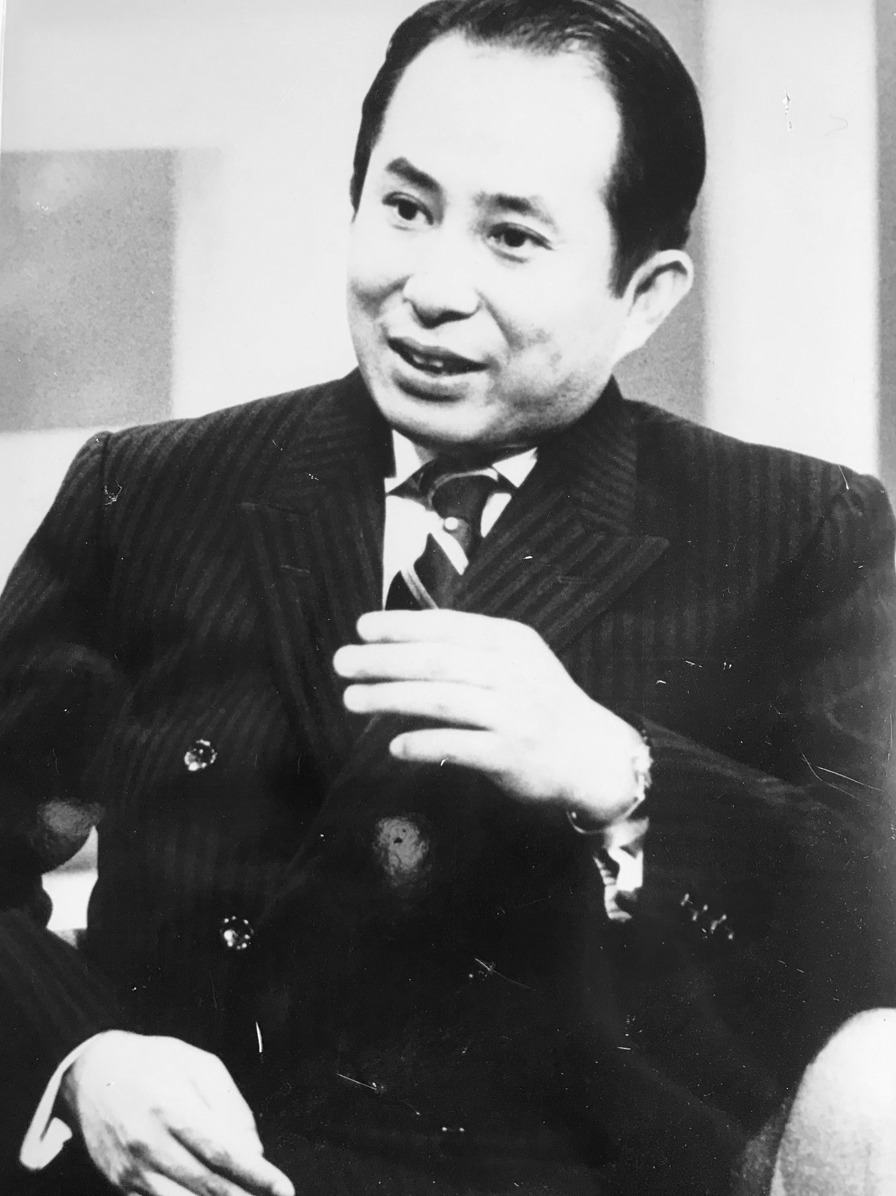
Dr. Hiromichi Nakajima

The greatest impact of the JHC has been in achieving wider access to therapeutic treatments for viral hepatitis. In 2004, pegylated interferon in combination with ribavirin was approved and integrated into the Japanese social health insurance system to treat chronic HCV. However, a co-payment rate of 30%, fixed in the social health insurance, obliged patients to make a substantial out-of-pocket payment and limited the number of patients who benefited from the therapy. In 2008, the powerful patient lobby, led by the JHC, helped to persuade the government to introduce special subsidies that reduced the out-of-pocket payment from US$ 7000 to US$ 1000 for a 48-week regimen. In 2010, the nucleos(t)ide analogues for chronic HBV infection also became eligible for the governmental subsidization, effectively ensuring more equitable access to the anti-HBV therapy. For hepatitis B immunization, Japan has opted since 1986 for targeted vaccination strategy, providing hepatitis B vaccine only to babies born to women identified as carrying HBsAg at the time of antenatal screening. This strategy has been effective in preventing mother-to-child transmission of HBV, but left most children susceptible to horizontal transmission. Following strong advocacy from the JHC and professional bodies, the government finally adopted universal infant hepatitis B vaccination in October 2016 [8]. Dr Nakajima, who unfortunately died in 1978 following hematemesis due to esophageal varices, could not see any of the success of the association he originally established.

Now, the JHC faces two serious problems: ageing of the population and decreases in membership. The mean age of association members now exceeds 70 years. The membership peaked in 2004 with over 10,000 members, and has since declined to 3500 members in 2020. This decline in membership may reflect the results of their successful historical patient-centered activities in Japan: a reduction in the number of new infections, and better access to effective treatment, both of which may limit the perceived benefits for young people living with viral hepatitis interested in joining these associations.

Nonetheless, the example of the JHC shows that the engagement of civil society is clearly critical to accelerating hepatitis elimination, as has been highlighted by a recent WHO survey for the Member States [9]. Japan’s unique experience sheds light on the importance of including the voice of people living with viral hepatitis in the decision-making process for ensuring equitable access to hepatitis services.

## Data Availability

Not applicable.

## Abbreviations

HBV: Hepatitis B virus
HCV: Hepatitis C virus
JHC: Japan Hepatitis Council
WHO: World Health Organization
